# A mouth watering case of *Mycoplasma salivarium* native mitral valve endocarditis: ‘case report’

**DOI:** 10.1186/s12879-023-08048-8

**Published:** 2023-02-07

**Authors:** Marc Scheen, Adrian Attinger, Tibor Huwyler, Mario Togni, Serban Puricel

**Affiliations:** Service de Cardiologie, HFR, Hôpital Cantonal de Fribourg, Chem. des Pensionnats 2-6, 1752 Villars-Sur-Glâne, Switzerland

**Keywords:** *Mycoplasma salivarium*, Infectious endocarditis, Oligoarthitis, Eubacterial PCR, Moxifloxacin

## Abstract

**Background:**

*Mycoplasma salivarium* is part of our commensal oral flora and readily resides in dental plaque. Although considered indolent, few case reports have documented its pathogenic potential in humans. To this day no case of *Mycoplasma salivarium* infectious endocarditis has ever been described.

**Case presentation:**

Our report describes a challenging case of *Mycoplasma salivarium* endocarditis, with a patient presenting with oligoarticular joint swelling, and later on in the course of his disease developed signs of right-sided heart failure. The diagnosis was initially mistaken for septic gonarthritis and was later established on the basis of echocardiography and eubacterial PCR of joint fluid.

**Conclusion:**

This report describes a first documented case of *Mycoplasma salivarium* culture negative endocarditis that was successfully treated with targeted antimicrobial therapy. Specific antimicrobial therapy targeting Mycoplasma spp, lead to clinical improvement, with radiological regression of the lesion and the resolution of the serum inflammation biomarkers.

## Background

*Mycoplasma salivarium* is an indolent bacteria, that is a part of our commensal oral flora and readily resides in dental plaque [1, 2]. It is not considered pathogenic, although reported cases of submasseteric abscess, and septic arthritis suggest otherwise [3–6]. To date, no case of *Mycoplasma salivarium* infective endocarditis has ever been described in the literature.

## Case presentation

A 69-year-old male patient, presented in mid-September 2018, with oligoarticular joint pain that progressed over a 3 week period and affected the right knee as well as the right elbow and the right shoulder. Symptoms were associated with morning stiffness lasting 30 min, as well as nocturnal pain. There was no history of trauma, upper respiratory tract infection or recent traveling abroad. There were no practice of risky sexual behaviour, and no recalling of any tick bites or animal encounters. His medical history is relevant for cutaneous psoriasis, type 2 diabetes mellitus, and Non-Hodgkin lymphoma in remission since 2012 after a hematopoietic stem cell autograft.

Upon presentation the patient had no fever. His right knee showed a spontaneous flexum with effusion made evident by a positive Flot and Glaçon sign. Left and right shoulder mobility were both limited. A left pre-pectoral Port-a-cath showed no signs of redness or swelling. The rest of the physical exam in the emergency department was unremarkable.

Initial biological work-up showed elevated CRP levels of 299 mg/dL, with mild leukocytosis. Serum chemistries and the coagulation panel were unremarkable. Blood cultures were also drawn upon admission, despite no signs of hyperpyrexia.

Arthroscopic drainage of the right knee was performed on the basis of suspected septic gonarthritis. Biochemical analysis of the synovial fluid revealed 50,000 elements with 64% neutrophils, without crystals. The patient was then started on an empirical antimicrobial regimen consisting of amoxicillin-clavulanate. Joint fluid cultures and blood cultures remained sterile after 14 days.

Over the course of the following days, the patients condition deteriorated, with fever and sequential multiple joint swelling involving the metacarpophalangeal joints of both hands, the proximal interphalangeal joints of the digits as well as the right elbow. Physical examination showed signs of right-sided heart failure with a new proto-systolic murmur of 3/6 intensity that was most prominent in the right second parasternal intercostal space. Bilateral pitting edema was present in the pre-tibial regions. Examination of the extremities revealed no signs of distal embolization or subcutaneous nodules. The neurological exam was normal.

Biological studies at week 2 showed worsening of the inflammatory biomarkers with a CRP level of 340 mg/dL, an erythrocyte sediment rate of 90 mm/h as well as normocytic hypochromic anaemia and thrombocytosis. The rest of the panel showed hyperferritinemia, a positive rheumatoid factor, as well as elevated liver enzymes. Anti-CCP antibodies were negative. Ultrasound of the small joints revealed various degrees of synovitis in the tarses, both wrists as well as the elbows.

**Fig. 1:**
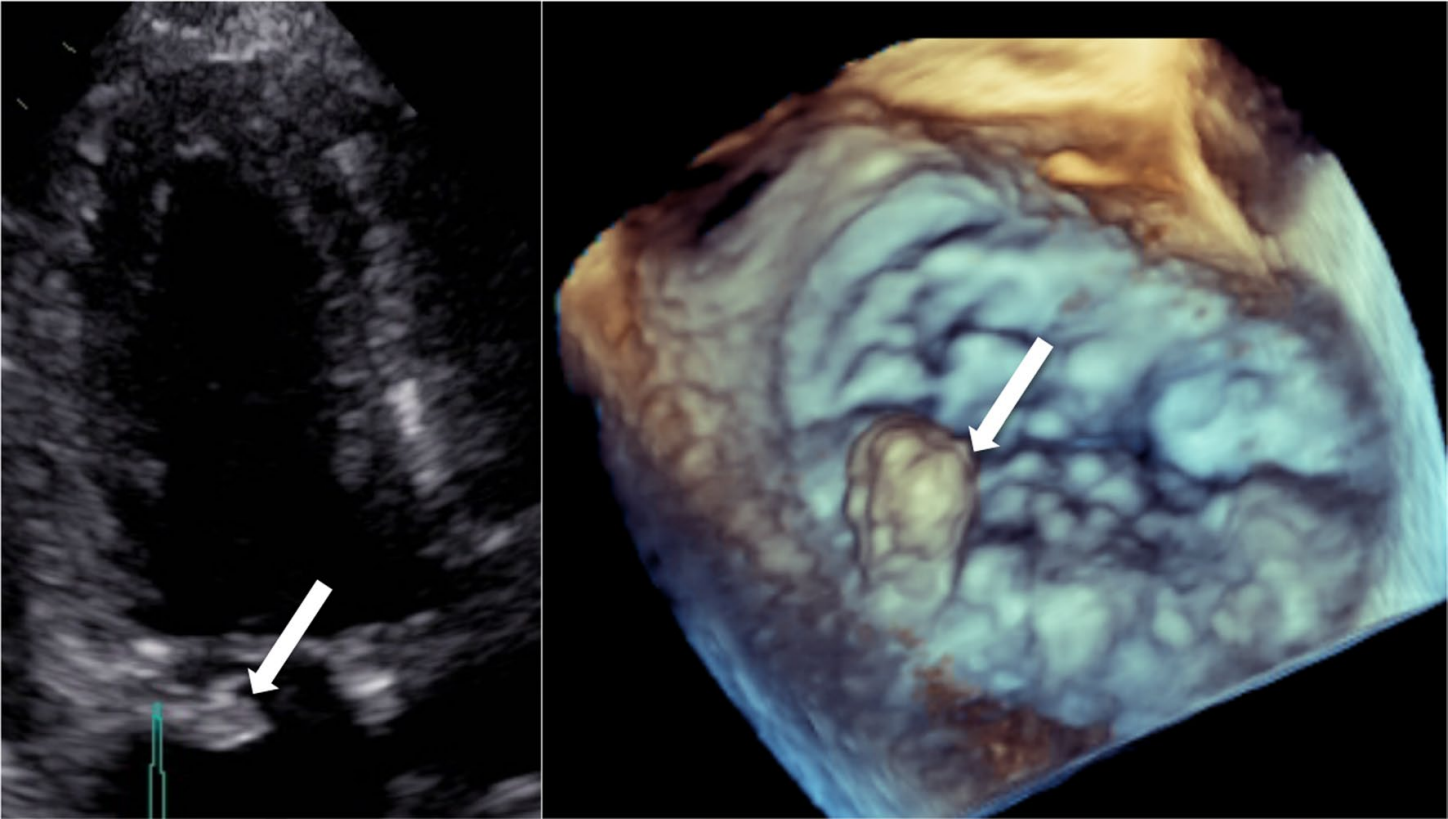
Transthoracic echocardiography showing 16 × 9 mm mobile vegetation of the posterior mitral valve leaflet

A 16 × 9 mm mobile vegetation of the posterior mitral valve leaflet, protruding into to the left ventricle without signs of left ventricular failure, septal abscess or mitral valve regurgitation was visualized on transthoracic echocardiography (Fig. 1). Extensive workup for endocarditis by means of multiple hemocultures, optic fundoscopy, cerebral imaging with CT and MRI, serologies for Coxiella burnetti, Brucella spp, Bartonella spp and Tropheryma whipplei, and antinuclear antibody panel with dosage of anti-phospholipid antibodies were all negative. The search for an oral cavity source of the endocarditis was both done clinically and through the use of an orthopantomogram. None were identified after thorough examination. A cervico-thoraco-abdomino-pelvic injected CT was performed and excluded a relapse of the NHL.

Eubacterial PCR of the synovial fluid sampled from the right knee during admission arthoscopy was performed retrospectively and came back positive for *Mycoplasma salivarium*. A transesophageal echocardiography was carried out 48 h later and confirmed the presence of the vegetation and revealed minimal pericardial effusion.

**Fig. 2:**
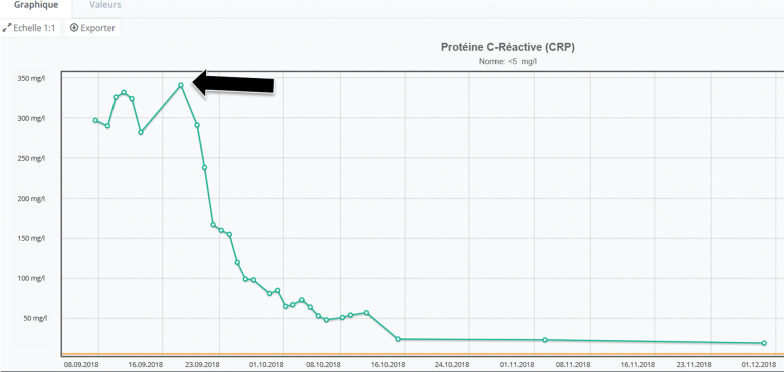
Drop of CRP levels after initiation of moxifloxacin (arrow)

Intravenous amoxicillin-clavulanate was replaced by IV ceftriaxone and oral doxycyclin. The antibiotic regimen was then further restricted to moxifloxacin as a final choice, after long QTc was excluded by 12-lead ECG. Figure 2 shows the evolution of C-reactive protein (CRP) after introduction of specific antimicrobial directed therapy. Full antimicrobial regimen was administered for a total of 6 weeks.

**Fig. 3:**
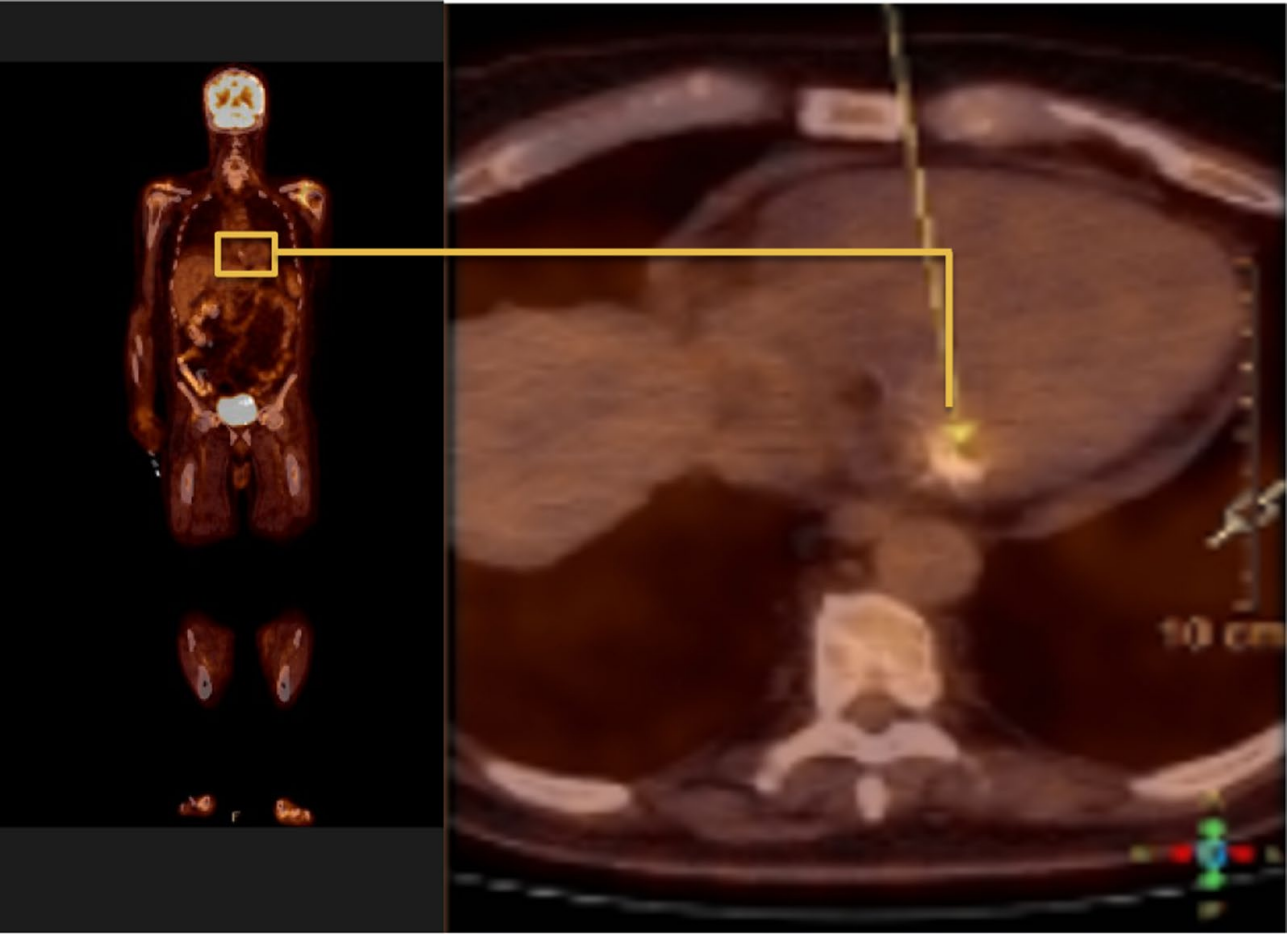
PET-CT hypermetabolic lesion of posterior mitral annulus

A follow up transthoracic echocardiography after 14 days of antibiotics showed almost complete regression of the vegetation. Distant embolisation given the rapid regression of the vegetation was excluded by means of cerebral MRI and a PET-CT. PET-CT imaging revealed a hypermetabolic foci on the mitral valve, comforting our initial diagnostic hypothesis of infective endocarditis (Fig. 3). The patient recovered completely after a long stand of antibiotic treatment. He continues to show persistent signs of remission to this day.

## Discussion and conclusions

This report exemplifies a difficult case of culture negative endocarditis, with a broad differential diagnosis that includes marantic endocarditis and atrial myxoma. To our knowledge, no documented human cases of *Mycoplasma salvarium* native or prosthetic valve endocarditis have been reported to this day. There are several case reports of *Mycoplasma salivarium* septic arthritis in the literature that occurred in immunocompromised hosts with either hematological malignancies or combined immunodeficiencies [4–6]. Our patient has a history of bone marrow transplantation and complete blood count showed lymphopenia upon admission. Although no causal relationship may be concluded from these series of cases, one may hypothesize that the relative immunodeficiency associated with our patients condition could have played a role in the developing of this invasive infection. Our paper further accentuates the importance of molecular diagnostic techniques such as eubacterial PCR, in identifying organisms that are either difficult to culture or whose infectious process has been altered by a course of antimicrobial therapy [7, 8]. Indeed, molecular diagnostic methods by means of DNA amplification such as eubacterial PCR are both sensitive and specific in diagnosing culture negative infections [7, 8]. While it is evident that there is no microbiological proof of imputability by means of blood or tissue cultures, *Mycoplasma salivarium* endocarditis remains the most likely diagnosis. This conclusion is based on the positive synovial eubacterial PCR combined with the positive response to specific antimicrobial directed therapy (moxifloxacin) as was shown by the complete regression of the mitral valve vegetation on follow-up transthoracic echocardiography. Retrospectively the initial choice of empirical antimicrobial therapy by means of intravenous amoxicillin-clavulanate alone is debatable. The choice of empirical antimicrobial therapy for suspected atraumatic, native joint septic arthritis in an immunocompromised patient with type 2 diabetes mellitus who is over 60 years old, has no history of risky sexual encounters and no available Gram stain should have a coverage of both Gram positive, Gram negative and an antipseudomonal spectrum. Either combined vancomycin with ceftazidime, cefepime or piperacillin-tazobactam until culture reception should have been our first choice in hindsight. The combination of daptomycin with either a carbapenem or a fluoroquinolone may be another alternative.

## Data Availability

Biological, echocardiographic, microbiological and PET-CT data have been made available to the editors. For further information please contact the corresponding author, Dr Marc Scheen (Marc.scheen@hotmail.fr).

## Abbreviations

PCR: Polymerase chain reaction
Spp: Species
CRP: C-reactive protein
Anti-CCP: Anti-cyclic citrullinated peptide
NHL: Non-Hodgkin Lymphoma
ECG: Electrocardiogram
MRI: Magnetic resonance imaging
PET-CT: Positron emission tomography computed tomography
DNA: Deoxyribonucleic acid
